# The reported effects of the COVID-19 pandemic on people with intellectual disability and their carers: a scoping review

**DOI:** 10.1080/07853890.2021.1922743

**Published:** 2021-06-10

**Authors:** Owen Doody, Paul M. Keenan

**Affiliations:** a Department of Nursing and Midwifery, Health Research Institute, University of Limerick, Limerick, Ireland; b School of Nursing and Midwifery, Trinity College Dublin, Dublin, Ireland

**Keywords:** COVID-19, pandemic, intellectual disability

## Abstract

**Background:**

People with an intellectual disability are a vulnerable group during COVID-19 due to multi-morbidity, frailty, underlying conditions/health problems, social circumstances and limitations in understanding. This places them at greater risk of more severe outcomes from COVID-19.

**Objective:**

To chart the evidence of the effects of the COVID-19 pandemic on people with intellectual disability and their carers reported in the research.

**Methods:**

A scoping review method was used to review literature published across eight databases in 2020 and included manual searches of reference lists of included articles.

**Results:**

Sixteen studies were selected for inclusion. The findings highlight that professionals, people with intellectual disability and their carers were keen to comply with pandemic related public health guidance. However, implementing infection prevention and control measures in certain contexts for people with intellectual disability was challenging. Thereby a rights-based, person-centred approach to care is essential and professionals need to extend their understanding and practice of collaborative working to include people with intellectual disability and their family/carers. The findings highlight that compliance with public health guidance and infection control measures can be difficult for people with intellectual disability to implement.

**Conclusions:**

There is limited good quality robust research on COVID-19 and people with intellectual disability and/or their carers experiences.

## Introduction

A pandemic is a worldwide epidemic in which a disease spreads easily and rapidly [1] and while rare they can be deadly. Pandemics occur when a virus exists and is easily spread from person to person in a population with little or no natural immunity [2]. On the 11th of March 2020, the WHO acknowledged the novel human coronavirus (COVID-19) as a pandemic and cases continued to rise globally. Throughout history, pandemics have occurred such as: Spanish flu (1918–1920), Asian flu (1957–1958), Hong Kong flu (1968), SARS (2002–2004), Swine Flu (2009–2010), MERS (2012–2016), Ebola epidemic (2014–2016) and Zika virus (1952–2015). However, the present pandemic COVID-19 has already proved to be a formidable foe [2].

People with multi-morbidity, frailty and underlying conditions are at increased risk of COVID-19 [3]. Public health response focussed on minimizing human-to-human spread to decrease secondary infections and infection control measures such as evading close contact with people with respiratory symptoms, engaging in good hand hygiene, cough etiquette practices [4]. In addition Governments implemented a variety of actions, including closure of non-essential services, travel limitations, cocooning and self-isolation to minimize interactions. COVID-19 is a serious health hazard to people across the world as it spreads rapidly [5] and up to 11th March 2021 there has been, 117,160,237 cases and 2,598,892 deaths reported [6]. People with intellectual disability are a susceptible group due to their need for support services and personnel [7] and are at increased risk of serious illness associated with COVID-19 due to health problems, social circumstances and limitations in understanding [8]. Challenges include access to understandable information about the disease; minimising the risk of infection; the risk of supports ceasing and the risk of increased anxiety, distress and behaviours that challenge [9]. The rapid changing environment and support structures can be difficult to accept as changes can result in distress due to confusion and disruption in their daily lives. Furthermore, carers often depend on support services for assistance and respite and the change to service provision affects carers well-being.

Public health actions to avoid transmission can increase the risk of loneliness and social isolation, which are well-recognized risk factors for physical and mental illness [10]. Attaining an understanding of the experiences of people with intellectual disability and their carers during COVID-19 is vital as both infectious diseases and actions to deal with them (e.g. quarantine and self-isolation) pose unique threats to their health and well-being [11]. This knowledge is essential if clear resources and supports are to be identified to manage such crisis in the future and to support people with intellectual disability and/or their carers, as often their voices are notably absent from research and discussions. Thus, this paper aims to review the current literature and identify key aspects relevant to managing COVID-19 to guide responsive and appropriate services in the future.

## Methods

A scoping review was selected as it allows for the evidence on a topic to be mapped, an examination of practice, policy and research, and the identification of gaps in evidence and policy [12]. Scoping reviews discover the nature and extend of the available evidence and plot the evidence by categorising the components of the literature, such as study design, sample, setting, intervention, underlying knowledge framework, aspects of relevance and findings. This creates a picture of the scope and significance of the literature and are used to determine literature on a specified area, recognise key thoughts, identify gaps in current literature and publicise outcomes [13]. The scoping review utilised Arksey and Malley [14] five-step process: (a) identifying the research question, (b) identifying relevant studies, (c) study choice, (d) plotting the data, and (e) arranging, summarizing, and communicating the outcomes. While these five steps are identified as a sequenced approach, it is in fact an interactive process where each step were revisited and progressed throughout the review. Results are expressed by way of a narrative in conjunction with tables and diagram illustrations [15].

### Identification of the research question

To meet the aim of this review, the authors addressed the following questions: (a) What effects of the COVID-19 pandemic on people with intellectual disabilities and their carers are reported? (b) What responses have been directed towards people with intellectual disability and their carers? and (c) What recommendations have been made regarding people with intellectual disability and their carers?

### Identification of relevant studies

To identify a breadth of literature a wide ranging set of keywords for search terms searching were adopted. Processes for searching integrated subject headings and Boolean operators were also used to expand and merge searches. Searches were completed using the Title OR Abstract and Keyword options, with individual searching of each search string and subsequent combination of all strings (Table 1). Inclusion and exclusion criteria were developed and applied (Table 2) and utilized to uncover relevant papers across eight electronic databases (CINAHL, Academic Search Complete, MEDLINE, PsycINFO, EMBASE, Scopus, Web of Science, Cochrane).

**Table 1.** Search terms.

S1	Intellectual disability* OR learning disability* OR developmental disability*
**S2**	Epidemic OR Pandemic OR COVID OR COVID-19 OR Coronavirus
**S3**	S1 + S2

**Table 2.** Inclusion/exclusion criteria.

Include	Exclude
Papers addressing information pertaining to coronavirus. Paper focus is on persons with intellectual disability and/or their carers. 01-01-2020 − 31-12-2020. English language. Primary peer reviewed research papers in journals.	Papers that do not addresses information pertaining to coronavirus. Papers where it is not possible to extract data focussing on persons with intellectual disability and/or their carers. Non-English language paper. Non-primary research such as reports, working papers/protocols, conference abstracts, government and non-governmental organisations documents, opinion papers, letters to the editor, discussion papers, correspondence papers

### Study selection

**Figure 1.**
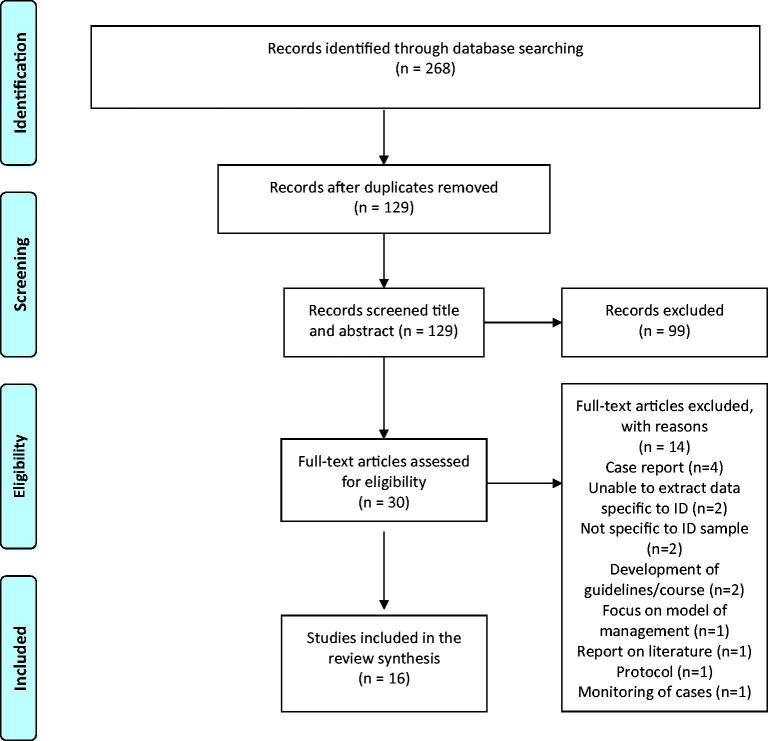
PRISMA 2009 Flow Diagram

Electronic database searches yielded 268 papers which were exported to Endnote and duplicates removed leaving 129 papers remaining. Following screening of title and abstracts guided by the inclusion and exclusion criteria 99 papers were excluded. 30 papers went forward to full text review and were assessed and agreed by the authors with 14 excluded and 16 included in the review. This review was limited to peer review research papers in 2020 in order to scope the evidence of the effects, responses and recommendations regarding COVID-19 and people with intellectual disability and their carers. All studies that met the inclusion criteria were included in this review as the authors wanted to capture all studies on COVID-19 within the intellectual disability population published in 2020. Furthermore, as critical appraisal and risk of bias are a choice in scoping reviews [16], the selection and reporting process followed [17] Preferred Reporting Items for Scoping Reviews (PRISMA-Sc-R) and PRISMA flow diagram [18] (Figure 1).

### Plotting the data

This scoping review aims to map the existing literature in terms of volume, nature, characteristics, and sources of evidence [19]. Plotting the data involved charting and extracting summaries from each paper and details of the extracted data are included in Table 3. Of the 16 included papers four were electronic surveys, four descriptive qualitative studies, three descriptive quantitative studies, two observational studies, one case series, one clinical study and one quasi experimental design.

**Table 3.** Data extraction table.

Author(s), Year, Title, Place	Aim and methodology	Population, methods	Summary of findings	Key messages	Limitations
[20] Mental Health of Parents of Special Needs Children in China during the COVID-19 Pandemic. China	To explore the mental health of parents of children with disabilities during the COVID-19 pandemic. Electronic survey.	1450 participants (parents of children with intellectual disability, autism, or visual/hearing impairment). 703 parents of children with intellectual disability, 454 with autism and 293 with visual/hearing impairment. Tools used – demographics, Behavioural Problems of Children during COVID-19, Psychological Demand of Parents during COVID-19, GHQ-12*, PSS*, PSI-SF-15*, NEO-FFI*. Data gathered - February 2020.	Ranked by mean mental health scores, parents of children with autism spectrum disorder (M = 2.88) were first, followed by an intellectual disability (M = 2.45) and a visual/ hearing impairment (M = 2.24). Mental health of parents of children with an intellectual disability was positively related to behavioural problems in children (*p* < .001), psychological demand of parents (*p* < .001), parenting distress (*p* < .001), parent–child dysfunctional interaction (*p* < .001), and di cult child (r = 0.26, *p* < .001); whereas it was negatively related to family support (*p* < .001), friend support (*p* < .001), and other necessary support (*p* < .001).	Behavioural problems of children were the main factors predicting mental health among parents. With quarantine there is limited access to education, rehabilitation, training, or intervention, and treatments services and this may lead to behavioural regression. Psychological demand of parents also predicts mental health among all parents. Parents are continually seeking out special treatment, medical equipment, and other services about how to deal with child’s behaviour, which would increase their psychological burden.	Cronbach’s alpha reported and above 0.7 for scales used. Examines three types of disabilities; thus, the results may not be generalisable. Self-report measures used. Online questionnaire may be limited to those with access and connectivity and literacy issues may affect response rate. Data collected very early in the pandemic and further data required to identify the long-term impact.
[21] Experiences and needs of direct support staff working with people with intellectual disabilities during the COVID-19 pandemic: A thematic analysis. Netherlands	To explore the experiences and needs of direct support staff during the initial stage of the COVID-19 lockdown. Descriptive qualitative study.	Eleven direct support staff from five intellectual disability services. Tools used - recorded weekly messages and thematic analysis. Data gathered – March/May 2020.	Four key areas 1) Emotional impact -The fear of infection, frustration and disappointment, sense of responsibility and overwhelmed with emotions. 2) Cognitive impact – coping, reflection, problem-solving attitude and perseverance 3) Practical impact - impact of preventive measures, experiencing time pressure, a lack of face-to-face, team meetings and increased use of digital consultation 4) Professional impact - cooperation and connectedness between support staff and other professionals.	Implications for policy and practice such as the provision of clear information from healthcare organisations and COVID-19 crisis teams is of paramount in assuaging staff and alleviating fears. It is essential that healthcare organisations emphasise the collective approach and support for direct support staff to overcome the sense of lone responsible and been in it on one’s own.	The small sample size in the study raises the question of whether the current findings can be generalised.
[22] A thematic analysis into the experiences of people with a mild intellectual disability during the COVID-19 lockdown period. Netherlands	To explore the experiences and needs of people with a mild intellectual disability with a (paid) job during the initial stage of the lockdown. A descriptive qualitative study.	6 adults with a mild intellectual disability. Tools used - semi-structured individual interviews and data analysed thematically. Data gathered – March/May 2020.	Increased levels of loneliness and enhanced health anxiety about the pandemic. Due to the preventive measures, the participants were no longer able to go to their work and undertake activities with their family, friends, or colleagues. As a result, there was a loss of structure and daily routine. In addition, there were the added difficulties of understanding and applying the new rules.	Need for more accessible information to be available to people with an intellectual disability such as easy read and accessible websites with up-to-date information about COVID-19. Consideration needs to be given as to how they could be supported to work from home and vitally important given the potential for additional waves.	Small sample size. All participants had a mild intellectual; disability and had voluntary work roles and may not be representative of wider intellectual; disability population.
[23] Changes in access to educational and healthcare services for individuals with intellectual and developmental disabilities during COVID-19 restrictions. United States of America	To capture changes in access to healthcare and educational services for individuals with intellectual and developmental disabilities that occurred shortly after restrictions were initiated. Electronic survey.	818 participants, (669 USA and 149 outside of the USA). Intellectual disability participants 649 (535-80% USA and 114-76.5% outside USA). Tools used - demographics, changes to educational services, changes to healthcare services and resources that caregivers found most helpful. Data gathered – April/May 2020.	Seventy‐four per cent of parents reported that their child lost access to at least one therapy or education service, and 36% of respondents lost access to a healthcare provider. Only 56% reported that their child received at least some continued services through tele‐education. Those that needed to access healthcare providers did so primarily through telemedicine. Telehealth (both tele‐education and telemedicine) were reported as helpful when available, and caregivers most often endorsed a need for an augmentation of these remote delivery services, such as 1:1 videoconference session, as well as increased access to 1:1 aides in the home.	COVID‐19 restrictions have greatly affected access to services for individuals with syndromic intellectual and developmental disabilities. Telehealth may provide opportunities for delivery of care and education in a sustainable way, not only as restrictions endure but also after they have been lifted.	Survey not validated against a clinical gold standard, nor did the researchers use any validated clinical measures or assessments alongside the survey. Online questionnaire may be limited to those with access and connectivity and literacy issues may affect response rate.
[24] Excess mortality in the first COVID pandemic peak: cross-sectional analyses of the impact of age, sex, ethnicity, household size, and long-term conditions in people of known SARS-CoV-2 status in England. United Kingdom	Aim to describe the rate of all-cause mortality throughout the first peak of SARS-CoV-2. Observational cohort study design with a cross-sectional analysis of people with SARS-CoV-2.	4,413,734 records of patients registered on the 11^th^ of May 2020 and having ≥1 year of health records. 55,951 patients had intellectual disability. Tools used - living in communal dwellings, SARS-CoV-2 exposure, socioeconomic and ethnic inequalities. Data gathered - May 2022.	People intellectual disability had a higher mortality odds, with the exception of diabetes and hypertension.	People with intellectual disability should be included on the list of groups who are more vulnerable to mortality associated with SARS-CoV-2 infection. There is a need for risk reduction strategies to reduce increased risk associated with SARS-CoV-2 for people with intellectual disability.	The use of clinical diagnostic codes (used to define the probable cases) is open to criticism.
[25] COVID-19 outcomes among people with intellectual and developmental disability living in residential group homes in New York State. United States of America	To describe COVID-19 outcomes among people with intellectual and developmental disability living in residential groups homes in the state of New. Quantitative descriptive study.	115 residential service providers with 20,431 residents and 19,453,291 general population. Tools used - number of residents, COVID-19 positive cases (confirmed by a physician), COVID-19 deaths, case rate, case fatality and mortality rate. Data gathered – May 2020.	COVID-19 case rates were substantially higher in residential group homes indicating a greater risk for this population group. The case rate was 4.1 times higher for people with IDD than for New York State. Case-fatality and mortality rates were markedly higher for people with IDD than for New York State: 15.0% compared to 7.9% for case-fatality; 7.8 times higher for mortality rates. Mortality rate for COVID-19 was higher for people with IDD residing in congregated settings.	Increased risk from COVID-19 for individuals living in congregate care settings due to the challenges these types of residence present to physical distancing. Need for public health surveillance systems to include disability status as a basic demographic characteristic. Need research to explore the specific impact of COVID-19 on people with intellectual and developmental disability and reduce their disproportionate risk in the current pandemic.	Reply on data provided by a coalition of organisations.
[26] COVID-19 outcomes among people with intellectual and developmental disability in California: The importance of type of residence and skilled nursing care needs. United States of America	To determine the impact of residential setting and level of skilled nursing care on COVID-19 outcomes for people receiving intellectual and developmental disability services, compared to those not receiving intellectual and developmental disability services. Quantitative descriptive study involving secondary data analysis.	354,640 receiving services 39,157,583 not receiving services. Tools used – California DDS data on COVID-19 outcomes for people with intellectual and developmental disability receiving services (number of deaths, type of service) Data gathered – May/October 2020.	Compared to those not receiving IDD services, those receiving such services had a 60% lower case rate, but 2.8 times higher case-fatality rate of COVID-19. COVID-19 outcomes varied significantly among Californians receiving services by type of residence and skilled nursing care needs: higher rates of diagnosis in settings with larger number of residents, higher case-fatality and mortality rates in settings that provided 24-h skilled nursing care. Diagnosis with COVID-19 among Californians receiving services appears to be related to the number of individuals within the residence, while adverse COVID-19 outcomes were associated with level of skilled nursing care.	When data is available, future research should examine whether these relationships persist even when controlling for age and pre-existing conditions.	The data currently available on COVID-19 outcomes among people with intellectual and developmental disability in the US is scarce. M data is available and efforts to understand and address how the pandemic is affecting this vulnerable population must make use of all available data.
[27] An audit of the well-being of staff working in intellectual disability settings in Ireland during the COVID-19 pandemic. Ireland – authors from the United Kingdom	To establish a baseline of the well-being of staff working in intellectual disability services in Ireland during the COVID-19 pandemic. Electronic survey.	285 staff in the Republic of Ireland. Tools used – demographics, CBI*, PHQ-9*, GAD-7*. Data gathered – May/June 2020.	Staff reported moderate levels of personal and work-related burnout and mild levels of anxiety and depression. Higher mean scores were recorded across scales from staff who worked in independent living settings and from staff who supported individuals with challenging behaviour. Staff working in home care felt the least supported by their employer.	Employers need to consider staff well-being, given the levels of personal and work-related burnout, and anxiety and depression. This is particularly true for staff who work in independent living settings and with adults with challenging behaviour. Future research should focus on proactive strategies for improving staff well-being in the short term, given the current resurgence of COVID-19 in Ireland.	The online self-selecting recruitment process may result in respondents who have higher levels of stress being more motivated to respond. No inferential analysis was undertaken and no pre-COVID-19 measures to compare.
[28] Examining the impact of COVID-19 in ethnically diverse families with young children with intellectual and developmental disabilities. United States of America	To investigate parental perspectives on the impact of COVID‐19 in a sample of predominantly Hispanic/Latinx, Spanish‐speaking families of young children with developmental delay or ASD living in the USA	77 parents’ preschool‐aged children (3–5 years old) with developmental delay or ASD. COVID‐19 interview (5 questions) via telephone. Data gathered March/May 2020.	The greatest challenge was around difficulties of being home during the pandemic such as being stuck at home and unable to leave the house, balancing work, caring for children and lack of childcare, changes in routine, emotionally supporting family, finding activities and preventing boredom for children. Parents reported financial concerns and dealing with significant challenges related to their child’s developmental services decreasing or stopping and feeling like they could not meet their child’s educational and developmental needs at home. Greatest concern was their family’s health and not getting COVID‐19 and dealing with their child’s behaviour problems. Some benefits reported such as, having more time together as a family, enjoyed a slower pace of life, gained more sleep, able to go outside more and meditate and reflect, communities coming together to support each other, they had learned to be more patient.	Both positive and negative aspects experienced. However, if restrictions continued for an extended period of time the pandemic would have a range of negative impacts on the family. Specifically, concerns about the long‐term impact of the pandemic on employment and finances and child’s emotional health. Families also expressed a variety of other long‐term concerns including lack of educational and developmental progress and emotional concerns for themselves and their child (fear of what the future will look like, feeling constant panic, getting very bored).	Findings present a snapshot of challenges faced by families with children with IDD but are from early in the pandemic and the long‐term impacts of the pandemic need consideration from people with IDD, families and professional’s perspective. Sample was ethnically, linguistically and socioeconomically diverse and caution must be taken in generalising the findings to other populations.
[29] Autism and COVID-19: A Case Series in a Neurodevelopmental Unit. France	Aim to describe the medical and behavioral manifestations of COVID-19 in individuals with autism and challenging behaviour. Retrospective case series.	16 participants with autism and ID. Clinical variables collected retrospectively (psychiatric symptoms prior to COVID-19 and COVID-19 symptoms. Data gathered – March/April 2020.	The main COVID-19 symptoms included upper respiratory infection, diarrhea, fatigue, fever and respiratory signs. One person’s epileptic seizures changed from partial to general.	Both common COVID-19 symptoms and idiosyncratic are manifested. A COVID+ unit for such patients is warranted and requires close collaboration with infectologists to limit both the spread of the virus and the ostracism of patients with autism.	Only 16 cases and 11 were COVID-19 confirmed cases. Took place in a specific unit for patients with autism and this type of support unit may not be available in other acute hospitals.
[30] COVID-19 deaths in people with intellectual disability in the UK and Ireland: descriptive study. United Kingdom	To understand if general population risk factors and comorbidities apply synchronously to the intellectual disability population. An observational descriptive study.	69 COVID-19-related deaths in intellectual disability from learning disability services in England and Ireland. Data gatered – March/June 2020.	The mean age of death (64 years) was younger than the general population and the cohort had high rates of moderate-to-profound intellectual disability (n = 43), epilepsy (n = 29), mental illness (n =29), dysphagia (n = 23), Down syndrome (n = 20) and dementia (n = 15). Considering mental health parameters, 11 had a diagnosis of severe mental illness and 18 having another mental illness. Just over a third had presented with challenging behaviour and a third had Down syndrome. 20 deaths in people with Down syndrome, 6 in people diagnosed with autism and 1 diagnosed with ADHD.	Reports on increased mortality and highlight the importance of exploring specific factors and comorbidities that may put people with intellectual disability at greater risk.	A case series collated through snowballing methodology may not be a representative sample. The use of psychiatrists for data collection may have caused high prevalence figures for mental illness.
[31] Impact of the initial response to COVID-19 on long-term care for people with intellectual disability: an interrupted time series analysis of incident reports. Netherlands.	To identify incident reports changed in a large care organisation from before until the end of the initial response phase. A quasi‐experimental interrupted time series design.	Weekly counts of incident reports, ranged from 6292 client incidents in 2016 to 6301 in 2020. Data gathered - September 2016/June 2020.	While overall the number of incident reports was stable across the years. The slope for the COVID‐19 period was significantly higher than for the pre‐ COVID‐19 period showing an increase in; reported incidents and incidents with aggression. Regression analysis showed a significant drop in medication error reports from pre‐COVID‐19 levels to the start of the COVID‐19 phase.	COVID‐19 measures may have increased compliance with health care protocols within more structured day routines, possibly leading to an actual decrease in medication errors.	Continued monitoring is needed to identify whether the rise in incidents flattened after this initial response phase and when scaling down of pandemic measures occur.
[32] Intellectual and developmental disability and COVID-19 case-fatality trends: TriNetX analysis. United States of America.	To analyze data from the TriNetX COVID-19 Research Network platform to identify COVID-19 patients.	30,282 patients of which 474 patients with developmental disability. Analysis focussed on trends in comorbidities, number of cases, number of deaths, and case fatality rate among patients who had a positive diagnosis for COVID-19.	The overall case fatality rate was comparable for those with IDD (5.1%) and those without IDD (5.4%). However, they identified much higher rates among adults under 75 year of age with an IDD than those without an IDD i.e. patients aged 18-74 years with an IDD 4.5%, without IDD 2.7%.; ages. They also reported people with an IDD had a higher prevalence of specific comorbidities associated with poorer COVID. These included those with endocrine, nutritional, or metabolic disorders and diseases of the circulatory system.	People with ID have higher prevalence of specific co-morbidities, such as hypertension, heart disease, respiratory disease, and diabetes, which are identified as risk factors for poor outcomes from COVID.	Comparisons between reports are difficult due to differences in governmental responses between countries, and differences in the types of samples.
[33] Impact of COVID-19 related lockdown on psychosocial, cognitive, and functional well-being in adults with Down Syndrome. Italy	To describe the impact of COVID-19 lockdown on psychosocial, cognitive and functional well-being in a sample population of adults with Down Syndrome (DS). Clinical study	46 participants with DS. Tools used - interRAI-ID, CPS*, ADLH*, IADLH*, DRS*, ABS*, SOCWD*, COMM*, PAIN scale and Visual Analog Scale. Data gathered – Pre (2015-March 2020) COVID-19 period April/May 2020.	The number of subjects that have worsened, improved or remained constant was significantly different for the IADLH scale. Regarding the pre-lockdown period, a significant worsening over time was found for the DRS score. Regarding the post-lockdown period, significant worsening in scores over time was found for the SOCWD scale, IADLH scale and DRS, while a significant improvement was found for ABS, ADLH, CPS and COMM scales.	Social isolation measures related to COVID-19 lockdown affect the functional and psychosocial well-being of adults with Down Syndrome. Lockdown should be considered a potentially traumatic life stressor event.	Study sample is small and pre-lockdown evaluations spread out over a large timeframe. Participants attended an outpatient clinic, therefore may have more complex health needs compared to the general DS population.
[34] Effect of the COVID-19 pandemic on the mental health of carers of people with intellectual disabilities. United Kingdom.	To identify the mental health of informal carers of children and adults with ID during the coronavirus pandemic and contextualise within the extent of their social support and stress/coping strategies. Electronic survey.	244 participants (carers of adults 107 and children 100 with ID and cares of children without ID 37). Tools used – demographics, Ways of Coping Questionnaire, Family Support Scale, GAD-7*, PHQ-9*, PHQ-ADS*, Short Defeat and Entrapment Scale, Objective Stress Scale. Data gathered – April/June 2020.	Support provided − 28% within the home, 64% remotely by phone (30%) or email/text (23%), videoconferencing (11%). Other support (28%) included outdoor conversations, social media and provision of supplies. Contact with professionals, only 3% were within the home with 90% provided remotely, 7% via videoconferencing. All groups made greater use of adaptive (Problem-Solving) coping strategies than maladaptive (Wish Fulfilment) coping strategies. Carers of children with intellectual disability reported significantly greater anxiety, depression, defeat/entrapment and wish fulfilment. Moderate to severe levels of anxiety were reported by 43% of carers of children with intellectual disability compared with 8% in parents of children without. Moderate to severe levels of depression, reported by 45% of carers of children with intellectual disability, compared with 11% of parents of children without. Carers of children with intellectual disability also received significantly less social support than parents of children without. Sources rated most helpful were partners, professionals and children; least helpful were neighbours, social/community groups and religious organisations. Carers of adults with challenging behaviour the least supported group.	The high scores of carers of children and adults with intellectual disability on these measures highlights that there should be a concern for their mental health and overall well-being. It is the overall burden of carer stress that impacts on carers’ mental health. Carer burden impairs mental health independently of the level of social support. Carers of people with intellectual disability, particularly those with challenging behaviour, reported that they received less support despite their greater needs.	Reliability of tools not reported. Comparison for the children group small 37 and no comparison group for the adult group.
[35] The use of online support by people with intellectual disabilities living independently during COVID-19. Netherlands.	To provide an insight into the use of online support during COVID-19. A retrospective, descriptive research design.	648 service users with intellectual disability had at least one contact with DigiContact support staff. Data gathered - first 20 weeks of 2019 / first 20 weeks of 2020.	The service dealt with a higher number of contacts per day during COVID‐19. The number of unplanned contacts per day per service user was significantly higher during COVID‐19 than during the first 11 weeks of 2020. COVID‐19 outbreak and related restrictive measures had quite an impact on the use of online support.	The sudden, substantial and temporary increase in unplanned online support indicates that people were considerably worried and experienced a high level of anxiety especially during the first weeks of the crisis. A lack of accessible information on the virus and the containment measures may contribute to the increase in unplanned online support use.	The study focussed on the first weeks of the COVID‐19 pandemic only. Would be useful to include data on the use of other online and onsite supports used.

Tools* - Activities of Daily Living Hierarchy (ADLH), Aggressive Behaviour scale (ABS), Cognitive Performance Scale (CPS), Communication Scale (COMM), Copenhagen Burnout Inventory (CBI), Depression Rating Scale (DRS), General Anxiety Disorder-7 (GAD-7), General Health Questionnaire-12 (GHQ-12), Instrumental Activities of Daily Living Hierarchy (IADLH), Neuroticism Extraversion Openness Five Factor Inventory (NEO-FFI), Patient Health Questionnaire (PHQ-9), Patient Health Questionnaire Anxiety and Depression Scale (PHQ-ADS), Parenting Stress Index-Short Form 15 (PSI-SF-15), Perceived Social Support (PSS), Social Withdrawal Scale (SOCWD).

### Arranging, summarizing, and communicating the outcomes

The final stage summarizes and communicates the findings. This scoping review generated 16 papers across seven countries (United States *n* = 5, Netherlands *n* = 4, United Kingdom *n* = 3, Ireland *n* = 1, Italy *n* = 1, China *n* = 1, France *n* = 1). The findings of the review are identified under the key questions identified in step one of the review process.

## Results

### What effects of the COVID-19 pandemic on people with intellectual disabilities and their carers are reported?

This review highlights that people with an intellectual disability and their carers are especially vulnerable to the physical, mental, and social effects of the pandemic. Such challenges include higher rates of mortality; more severe health outcomes; substantial reduction in the availability and access to face-to-face services; minimizing the risk of infection; risk of increased mental illness, such as loneliness, agitation, anxiety, distress and in some cases increased challenging behaviour. From the perspective of people with intellectual disability there is evidence that there is a higher risk for those residing in residential group homes [24], of developing more severe outcomes from COVID-19 [31], higher co-morbidities [30,31] and much higher mortality [23,24], particularly among adults under 75 years of age [31] as compared to the general population. Diseases of the circulatory and respiratory system were high along with endocrine, nutritional and metabolic disorders [31]. The main COVID-19 symptoms included upper respiratory infection, diarrhoea, fatigue, fever and respiratory signs [28]. The most common underlying physical health conditions were diagnosis of epilepsy, dysphagia, dementia, hypertension, asthma and diabetes [29]. However [25] identifies that while those receiving intellectual disability services had a lower-case rate, they had a higher case-fatality rate of COVID-19. As a result of COVID-19, there was a loss of structure and daily routine [22] creating challenges around being stuck at home and unable to leave the house, finding activities and preventing boredom [27]. However, some benefits were reported such as, having more time together as a family, enjoyed the slower pace of life, gaining more sleep, learned to be more patient and communities coming together to support each other [27]. A reduction in service and societal demands has also led to some people with an intellectual disability being much more settled and content in their lives [33].

From a family perspective, parents greatest concern was their family’s health, not getting COVID‐19 and dealing with their child’s behaviour problems [27]. Financial concerns, managing significant challenges related to their child’s services decreasing or ceasing and feeling like they could not meet their child’s needs at home impacted on their mental well-being [27]. Of note was that families caring for children with intellectual disability reported significantly greater anxiety, depression, defeat/entrapment and wish fulfilment [33] and their mental health was positively related to behavioural changes [20]. Additional family demands, a loss of routine, educational and therapeutic supports and the introduction of new stressors [22,27] all resulting in a decreased capacity to cope and increased isolation [35]. On the other hand, staff reported moderate levels of personal and work-related burnout and mild levels of anxiety and depression [26]. Staff working in independent living settings and individuals with challenging behaviour report highest level anxiety, and this was related to incidents with aggression [30,32]. Of note was that staff in home care felt least supported by their employer [26] and in residential care settings a drop in staff medication errors from pre‐COVID‐19 levels was noted [30]. Furthermore, from a staff perspective, they identified that Covid-19 had an impact across all domains of their life at an emotional level there is a fear of infection, a sense of frustration, disappointment and responsibility that can lead to a sense of been overwhelmed by emotions [21,35]. At a cognitive level staff were challenged by COVID-19 in terms of coping skills, reflection, problem-solving skills, attitudes and perseverance [35]. From a practical perspective, preventive measures-imposed limitation for people with intellectual disability and resulted in staff experiencing; time pressure, a lack of face-to-face contact and a demand to be able to use and access digital technologies [35]. Furthermore, from a professional perspective, there was a loss of connectedness with other professional and support staff [35]. These factors all increased the sense of isolation, exacerbated the sense of anxiety about COVID-19 and added to the difficulties in understanding and applying the new rules [21].

At a service level COVID‐19 had quite an impact on service delivery resulting in many of them closing, reducing service access and/or going online and providing one to one home support [22,27]. Services reported dealing with a higher number of unplanned online contacts per day during COVID‐19, indicating that people were considerably worried and experienced a high level of anxiety especially during the first weeks of the crisis [34]. Within some residential services, incident reports reduced overall as a result of lockdown measures, however incidents of aggression increased [30]. This review highlights a paucity of data on COVID-19 trends among the intellectual disability population, variations and limitations in recording processes utilised and little evidence of interventions utilised to support people with intellectual disability and their families [23,24,28,29,31].

### What responses have been directed towards people with intellectual disability and their carers?

The COVID-19 pandemic has affected all aspects of the intellectual disability community and particularly posed an increased risk for individuals living in congregated care settings due to the challenges these types of residence present regarding physical distancing [24]. A COVID-19 unit was one clear measure taken to limit the spread of the virus [28]. This response was a collaboration between staff, service providers and infectologists. However, for those in the community there was generally a lack of accessible information on the virus and the containment measures leading to a demand for support addressed *via* online support [21,34]. As quarantine limited access to education, rehabilitation, training, or intervention and treatment services, behavioural regression were evident [20]. Leading to greater use of adaptive (problem-solving) coping strategies rather than maladaptive (wish fulfilment) coping strategies [35]. In addition, from a staff perspective greater compliance with health care protocols and more structured day routines, lead to a decrease in medication errors [30].

Evident with the findings of this review was that there is a paucity of data on COVID-19 trends among the intellectual disability population which exposes the reality that there is no adequate surveillance structure in place to monitor COVID-19, or other public health outcomes, among the intellectual disability population [31]. In the absence of such data there is a risk of a potential under-reporting of cases and related deaths and measures targeted at the general population should not be assumed to be sufficient for the intellectual disability population [29]. Thereby medical practitioners should be aware of the increased risk for individuals with intellectual disability, even mild symptomatology, and need to accurately code deaths of those who die from COVID-19 [24]. Adverse COVID-19 outcomes were associated with the level of skilled nursing care, where low levels of skilled care resulted in higher levels of adverse effects [25].

Response of external agencies such as schools and community teams to providing continued support, albeit remotely is mixed with some agencies attempting to provide continued support but others providing little if any assistance. Those that needed to access healthcare providers did so primarily through telemedicine [22]. Telehealth (both tele‐education and telemedicine) were found to be helpful, and often there was a need to augment these with 1:1 videoconference session [22]. Other supports included outdoor conversations, social media, provision of supplies and contact with professionals [33]. Telehealth supported the delivery of care and education in a sustainable way, during and post restrictions [22]. Within the literature, the sources rated as most helpful were partner/spouse followed by professionals; however, carers of adults with mild challenging behaviour were the least supported [33]. Social isolation measures affect the functional and psychosocial well-being of people with intellectual disability and lockdown should be considered a potentially traumatic life stressor event [32]. Given that COVID‐19 restrictions have greatly affected access to services for individuals with intellectual disabilities, [22] the provision and use of online support has been a key service development [34].

### What recommendations have been made regarding people with intellectual disability and their carers?

The literature reviewed clearly highlights that healthcare professionals need to be aware that people with intellectual disability are a high-risk population [23,25] and should be aware of the potential for severe outcomes [31]. This risk is also further increased for individuals living in congregate care settings due to the challenges these types of residence present for physical distancing [24]. All family members, care workers, and people with intellectual disability need to take extra precautions to ensure their health and safety during the COVID-19 [24]. Service leaders to recognise the need for extra supports such as social stories to explain the wearing of PPE [25] and the use of technology to provide virtual visiting and telemedicine systems.

Given the paucity of data on COVID-19 regarding the intellectual disability population, there is a need for surveillance structure to be put in place to monitor COVID-19 and other health outcomes [31]. This has implications for policy and practice such as the provision of clear information from healthcare organizations and COVID-19 crisis teams [35]. Such an approach requires public health surveillance systems to include disability status as a basic demographic characteristic [24]. People with intellectual disability should be included on the list of groups who are more vulnerable to mortality associated with COVID-19 [23] and there is a need for more accessible information for people with an intellectual disability such as easy read and accessible websites with up-to-date information about COVID-19 [21]. Furthermore, there is a need for risk reduction strategies to address increased risk associated with COVID-19 for people with intellectual disability [23].

Given the unpredictable and uncertain future the consequences of the disruption to all services and day-to-day life and loss of structure and routine raises anxiety for people with intellectual disability. There is a clear need for improving psychological support and identifying psychological intervention with a set of activities that can be safely implemented [24]. There is a need to consider the impact of the COVID-19 experience on the mental health and overall well-being of people with intellectual disability [33] and thus explore the specific impact of COVID-19 on people with intellectual disability and reduce their disproportionate risk [24]. In addition, consideration needs to be given as to how people with intellectual disability can be supported to work or be educated from home given the potential for additional waves of the virus [21].

The important role of families and carers [24] needs to be recognized and policies regarding family/visitor access need to be addressed, as restrictions over an extended period have a range of negative impacts on the family [27]. Families concerns regarding a lack of educational and developmental progress and emotional concerns need to be considered [27]. For staff, a collective approach to support direct support staff to overcome the sense of lone responsibility is required [35]. Moreover, employers need to consider staff well-being, given the levels of personal and work-related burnout, and anxiety and depression [26] and research should focus on proactive strategies for improving staff well-being in the short term, given the current resurgence of COVID-19 [26].

## Discussion

This review and the wider literature highlights that people with intellectual disability are at increased risk and vulnerable to infection, heightened if the person resides in a congregate setting [10,36–38]. In addition, it is difficult to ensure adequate physical, or social, distance as they often depend on physical contact with a professional or career for their daily activities and care needs and find it difficult to understand the new restrictions [39,40]. The greater the severity of disability the greater the need for support and thus the greater the risk. These factors increase the possibility of transmission of infection leading to complications such as pneumonia, acute respiratory distress and organ dysfunction [41]. Consequently, people with intellectual disability are likely to experience more severe cases [31] and as a result have poorer outcomes [42] resulting in death [43]. Given the increased likelihood of infection and comorbidities among people with intellectual disability they are likely to suffer more serious clinical outcomes [40] and experience higher fatality rates [44,45] which is double that of the general population [31]. One could expect these greater risks given the pre-existing health disparities faced by people with intellectual disability prior to the pandemic, including poorer health outcomes [46], limited access to health care services [47], participation in fewer prevention and health promotion activities [48], increased risk for chronic health conditions [46] and earlier age of death when compared to the general population [49]. However, despite the increased risk and health disparities, only 2% of people with intellectual have been reported as having a COVID-19 test [50].

Of concern within this review is the lack of surveillance or data collection systems which are needed to assure the right types of health protection actions (prevention, treatment, and mitigation). Population-based registries have an important role in determining the prevalence, aetiology, distribution, frequency and severity of a particular condition/disability in a national or international region. However, there is little if any population-based data on people with intellectual disability and there needs to be an identification of people with intellectual disability in a systematic manner. Without the capacity to identify people with intellectual disability in emergency management surveillance systems, they may be overlooked when interventions are planned and evaluated. While this review highlights that infection control measures in healthcare facilities have managed the pandemic well there is little known about infection control knowledge, attitudes, and practices among staff [51]. This is essential as care environments have special infection control needs as they act as both the individual’s permanent home and place where medical care is provided [52]. Evident within this review is that although there has been an increase in COVID-19 publications in intellectual disability there is an absence of the voice of the person with intellectual disability in such publications [53]. This absence needs to be considered as we look to the long-term impact and implications of Covid-19 regarding public health guidance, isolation, cocooning, withdrawal of services, family stress on individual’s psychological, mental and physical well-being. This absence may currently be addressed and newer publications may have this as their focus such as [54] or nested within a study. Furthermore, as this review only covered the year 2020, there is no information on vaccination and monitoring of vaccination uptake is required. It is important that the feelings, experiences, awareness and the unique concerns of people with intellectual disability and their families should be prioritised and not overlooked, forgotten or neglected [55,56]. Furthermore, research is needed on strategies of improving the health protection measures for people with intellectual disability during the outbreak [57] and identify resilience measures [58].

This review highlights the psychological and social consequences of the pandemic for people with intellectual disability who have stopped participating in their communities and have had their routines interrupted during lockdown/restrictions for a prolonged period [59,60,61] and access to education, health, vocation and mental health service disrupted [50,62]. In addition, many have found it difficult to understand COVID-19 and self-regulate their behaviour, creating a risk of behaviours that challenge which negatively affect the emotional well-being and quality-of-life of the person [63,64]. One could argue further segregation is occurring not just by the wider society or generic health services, but also in terms of service provision and this intensifies the sense of isolation. Thus, services and society need to strike a balance between humanity and restrictions, as for many, proximity to skilled caregivers and loved ones is required to bridge gaps in intellectual and communication abilities and to make day-to-day life fulfilling and manageable. This humanity and rights debate is further emphasised in healthcare decision-making, where there are strong ethical justifications for robust triage systems when capacity and resources to provide critical care to patients are overwhelmed [65]. However, there are also difficult ethical dilemmas and decisions as to which COVID-19 patients should go on life-saving treatment because of the shortage of medical equipment [66] and accounts of using disability as a factor in denying life-saving treatment are discriminatory [67]. This raises serious concerns regarding disability and human rights [68] and some reports highlight vulnerable populations, including people living with disabilities, being spoken about as if they were disposable or expendable [69].

This review highlights the essential and important role families play in the support, health and well-being of individuals with intellectual disability [70]. With public health guidelines emphasising maintaining a 2-meter distance to fight the virus, families face growing challenges that may be impossible to meet [71], and it may be unreasonable to expect a person with intellectual disability in care to cope when separated from their family [72]. Families caring in their own home are experiencing higher levels of concerns and anxiety [33,73,74] which is fuelled by a loss of routine, educational and therapeutic supports and the introduction of new stressors [27]. These all result in a decreased capacity to cope and increased isolation for the family [35,75] and despite some proactive approaches to use alternative communication means to provide support services they may not be effective in filling the gap created by reduced face-to-face contact [76]. In addition, electronic service provision needs to be evaluated and while they have shown benefits and effectiveness, implementation and integration within health systems, and user satisfaction [77,78] concerns have also been raised regarding screen time and the long-term impact of service reduction [27]. While telehealth/electronic service can show positive outcomes [79], there are issues with access, accessibility and support for usage [80,81]. As we face into 2021 and with the advent of a vaccine the question for people with intellectual disability and their families is, will they be identified as essential and prioritised for vaccination? A key issue for families is that if they as a direct family carer fall ill, can the health system be inclusive of the needs of individuals with intellectual disability [82] and provide a substitute caregiver, who is aware of the needs of the person and can this be put in place until recovery of the usual caregiver [83].

## Limitations

As the focus of this review was to map the evidence, no evaluation of the quality of evidence/quality appraisal were performed and this paper only provides an explanatory account of available information. This review only included papers published in English and this may have resulted in the exclusion of relevant papers. In addition, the review only covers the year 2020 and we acknowledge that the evidence regarding COVID-19 grows daily. However, within the review, the authors engaged with relevant papers post 2020 for inclusion within the discussion. Furthermore, this review has to be considered within the limitations identified within the studies that met this paper’s inclusion criteria mainly the fact that (1) most data collection took place very early in the pandemic and further data is required to identify the long-term impact of Covid-19, (2) samples were small in size and not representative of the full nature of the intellectual disability population, (3) data collection utilised self-reporting measures and were administered mainly online where there may be limited issues with access, connectivity and literacy and (4) the data collection tools used were not validated, inferential analysis was not conducted and no pre-COVID-19 measures were available to compare against. These factors all affect the reliability, validity and generalizable of the studies in this review and further analysis as to whether these aspects are addressed in 2021 studies is warranted.

## Conclusion

This review highlights that the peer-reviewed press is dominated by professional opinion pieces, editorials and letters to the editors presenting various personal as well as professional polemics, some of which are supported with reference to the literature, as well as anecdotal evidence. However, there is limited research about pandemics/epidemics and people with intellectual disability. Rarely is space provided for population studies or the rich perspectives of the experiences of the person with an intellectual disability. In saying that, the research that does exist clearly highlights that core to the management of pandemics and epidemics are 1. A rights-based, person-centred approach to care is essential as professional and organisational approaches, as well as ubiquitous strategies often fail or fall short; 2. Professionals need to extend their understanding and practice of collaborative working to include people with an intellectual disability and their family/cares, not just begin to include other professional colleagues; 3. High levels of concordance with public health guidance and 4. Rigorous implementation of quality infection control measures are essential prerequisites.

## Data Availability

No data are available other than that reported in this review and available in original published papers used in this review.
